# Rapid thawing of frozen bull spermatozoa by transient exposure to 70 °C improves the viability, motility and mitochondrial health

**DOI:** 10.1590/1984-3143-AR2022-0127

**Published:** 2023-10-20

**Authors:** Hai Thanh Nguyen, Son Quang Do, Rukmali Athurupana, Takuya Wakai, Hiroaki Funahashi

**Affiliations:** 1 Department of Animal Science, Graduate School of Environmental and Life Science, Okayama University, Okayama, Japan; 2 Department of Animal Production, Faculty of Animal Science and Veterinary Medicine, Nong Lam University, Ho Chi Minh City, Vietnam

**Keywords:** bull semen, cryopreservation process, phospholipase C zeta1 (PLCZ1), temperature of thawing

## Abstract

Up to now, the definitive conclusion of the positive effects of rapid transient thawing at higher temperatures for shorter durations has not been obtained yet and is still under discussion due to some contradictory findings and limited assessment of post-thawed parameters. The purpose of the current study was to evaluate the effectiveness of rapid thawing in water at 70 °C by using various post-thawed parameters of frozen bull spermatozoa. Experiment 1, monitoring the change of temperature inside frozen bull straw thawed in water at different temperatures. Experiment 2, evaluation of various post-thawed characteristics of frozen bull spermatozoa thawed in water at different temperatures by using a computer-assisted sperm analysis, flow cytometry and immunocytochemistry. The time it took for the temperature inside the straw to warm up to 15 °C was nearly twice as faster when the straw was thawed in 70 °C water compared with 39 °C. Although there were differences among bulls, viability, motility, and mitochondrial membrane potential of spermatozoa thawed at 70 °C for 8 seconds and stabilized at 39 °C for 52 seconds were significantly higher than those of controls (thawed at 39 °C for 60 seconds) at 0 and 3 h after thawing. Just after thawing, however, there were no differences in acrosome integrity and distribution of phospholipase C zeta1, whereas mitochondrial reactive oxygen species production was significantly lower in spermatozoa thawed at 70 °C. From these results, we conclude that rapid thawing at 70 °C and then stabilization at 39 °C significantly improves viability, motility and mitochondrial health of bull spermatozoa rather than conventional thawing at 39 °C. The beneficial effect of rapid transient thawing could be due to shorter exposure to temperatures outside the physiological range, consequently maintaining mitochondrial health.

## Introduction

Cryopreservation of bull semen has contributed significantly to the genetic improvement and efficient reproduction for decades in the dairy and beef production industries, but a low fertilizability of frozen-thawed spermatozoa seems to be still the main concerns (Upadhyay et al., 2021). It is recommended that frozen bull semen straws are thawed under the physiological temperature, even though slow thawing of frozen spermatozoa seems to promote recrystallization and consequently damages the organelles (Sharafi et al., 2022). Although thawing at higher temperatures for shorter durations has been tried with better results for some important parameters to overcome a low quality of frozen-thawed buffalo (Rastegarnia et al., 2013) and cattle spermatozoa (Lyashenko, 2015), due to some inconsistent results (Yilmaz et al., 2019), the definitive conclusion has not been obtained yet and is still in discussion.

In the previous reports mentioned above (Rastegarnia et al., 2013; Lyashenko, 2015), motility and viability of spermatozoa have already been assessed after thawing at 70 °C for 6-7 seconds, but it is not yet clear if there are any differences among bulls in the effect of thawing by rapid thawing on frozen sperm quality. Additionally, since other crucial parameters associated with mitochondrial function, such as mitochondrial membrane potential (MMP) and mitochondrial reactive oxygen species (ROS), have not also been made clear yet. The effectiveness of rapid thawing at a high temperature has never been discussed with evidences of mitochondrial activity, oxidative stress and others. Mitochondrial membrane potential is well known to be positively correlated with the viability, motility, and fertilizability of spermatozoa (Marchetti et al., 2012). An excessive ROS level during the freeze-thawing process could impair plasma membrane, mitochondria homeostasis, motility, and consequent penetrability (Bahmyari et al., 2020). Furthermore, cryopreservation is known to significantly affect phospholipase C zeta1 (PLCZ1), one of the PLC family members universally recognized as a potential candidate responsible for resumption of oocyte meiosis at fertilization (Amdani et al., 2015; Thanassoulas et al., 2022), and consequently fertility (Moreau et al., 2019).

Therefore, the aim of the present study was to investigate the effects of thawing of frozen bull spermatozoa at different temperatures (39 and 70 °C) and subsequent stabilization at 39 °C on the sperm quality parameters and PLCZ1 distribution.

## Methods

### Chemicals and frozen bull semen

All chemicals used in the study were purchased from Sigma-Aldrich Japan G.K. (Tokyo, Japan), unless specified otherwise. The medium used for manipulating and incubating sperm was modified TL-HEPES-PVA solution (Akaki et al., 2009).

Frozen semen collected from four Japanese Black Bulls (3-8 years old) with excellent fertility scores and prepared for commercial purpose in 0.5 mL straws was donated from a local public AI center, Okayama Prefectural Center for Animal Husbandry and Research. At the center, bull semen was collected from bulls by using the artificial vagina according to a standard protocol authorized from point of view on animal welfare and ethics. The collected semen was processed and frozen by using a protocol (Hamano, 2016). Briefly, semen was mixed with extender solution (112.7 mM tris(hydroxymethyl)aminomethanol, 39.7 mM citric acid, 20.8 mM fructose, 43.8 mM lactose and 53.5 mM raffinose containing 20% (v/v) egg yolk) and cooled to 4 °C in refrigerator. After the concentration of glycerol in the extender solution was gradually increased to 6.5% (v/v) at 4 °C, the diluted semen was filled in 0.5 mL straws. The straws were frozen by cooling to -7 °C in 1-2 min and then to -80 °C in 3 min before being thrown into liquid nitrogen. No ethics approvals were required because only commercial frozen semen was used in the current study.

### Evaluation of spermatozoa quality

#### Flow cytometry

Sperm suspension was analyzed for viability, MMP, acrosome status, and ROS by using a Gallios flow cytometer (Beckman Coulter Inc., Brea, CA, USA) after repeatedly washing before and after staining with specific dyes to remove non-sperm particles which can affect our results. The flow cytometer was equipped with three argon lasers and a total of ten fluorescence channels (five 488 nm, three 638 nm, and two 405 nm). Green fluorescence emissions were detected in 525 nm band pass filter, as well as orange fluorescence wavelength in 575 nm band pass detector, and red fluorescence in 620 nm filter. For each sperm suspension sample, one test tube containing 0.5 mL of the diluted sperm suspension at 5x10^6^ cells/mL was acquired the result of 10,000 events per replicate at forward and side scatter channels. The other non-sperm events such as debris (Rodriguez et al., 2012; Gürler et al., 2016) or alien particles (Petrunkina et al., 2010) were also excluded from the cell population of interest by gating out on the basis of the forward and side scatter dot plots. The analysis of flow cytometry data was performed by using Kaluza analysis software (Beckman Coulter Inc., Brea, CA, USA).

Viability of spermatozoa was determined according to the commercial protocol of a LIVE/DEAD viability kit (Thermo Fisher Scientific Inc., Waltham, MA, USA). Sperm samples were incubated with SYBR safe DNA (100 nmol/L) and propidium iodide (PI) (10 µmol/L) at 39 °C for 1.5 min in dark (Athurupana et al., 2015; Gonzalez-Castro et al., 2019). The viability was determined by the proportion of SYBR+/PI- gates.

Sperm MMP was assessed by using JC-1 (5’,6,6’-tetrachloro-1,1’,3,3’-tetraethyl benzimidazolyl-carbocyanine iodide) by which forms aggregates making an orange fluorescence at 590 nm (high MMP) and monomers giving a green wavelength at 527 nm (low MMP). Sperm suspension was incubated with JC-1 (0.76 µmol/L) at 39 °C for 8 min in dark (Athurupana et al., 2015; Macias-Garcia et al., 2012). The percentage of spermatozoa with high MMP was calculated by the ratio of cells in orange fluorescence gate.

Acrosome integrity (intactness of acrosome membrane) was evaluated by using PI and fluorescein isothiocyanate-conjugated peanut agglutinin (FITC-PNA). Sperm samples were incubated with PI (10 µmol/L) and FITC-PNA (1 µg/mL) at 39 °C for 8 min in dark (Athurupana et al., 2015; Gurler et al., 2016). The percentage of spermatozoa with intact acrosome membrane was calculated by the ratio of cells in PNA- gates.

For ROS detection, sperm suspension was incubated with PI (10 µmol/L) and highly sensitive DCFHDA reagent (Dojindo Laboratories, Kumamoto, Japan) diluted at 1:1,000 with loading buffer for 30 min at 39 °C in dark (Gonzalez-Castro et al., 2019). The intracellular ROS levels in the total spermatozoa and the only live subpopulation were determined.

### Motility of spermatozoa

A computer-assisted sperm analysis system with 60 digital images per second (Sperm Motility Analysis System, Digital Image Technology, Tokyo, Japan) was used to determine percentages of the total, progressive and rapid progressive motile spermatozoa (%), and kinetic parameters (Ogata et al., 2022; Sapanidou et al., 2022), such as the velocity straight line (VSL), velocity curved line (VCL), velocity average path (VAP), amplitude of lateral head displacement (ALH), and beat cross frequency (BCF). The set-up of the system used for conventional motion characteristics of bull semen was according to the manufacturer’s guidelines basically based on the WHO criteria (World Health Organization, 1999, 2010) previously described in more specifications for bull semen (Gürler et al., 2016; Bulkeley et al., 2021) (Spermvision standard bulls, average orientation change <2.5, straightness <0.5, linearity <0.35, distance straight line <4.5, distance average path/radius ≥3 and linearity <0.5). Frozen-thawed sperm suspension was centrifuged once (700×g, 3 min, 39 °C) and diluted with TL-HEPES-PVA (1x10^7^ cells/mL). The suspension was analyzed in a Makler counting chamber (FUJIFILM Irvine Scientific, Santa Ana, CA, USA) at 39 °C, according to the sperm analysis system’s procedures. A minimum of 300 spermatozoa from 3 different microscopic fields were analyzed by the system.

### Distribution of PLCZ1 in spermatozoa

Indirect immunocytochemistry was performed according to a protocol described previously by our laboratory (Ioki et al., 2016). In brief, after air-drying and fixation with methanol, sperm samples on glass slide were incubated in blocking solution, TL-HEPES-PVA containing 5% (w/v) BSA, for 1 h at 37 °C to block the nonspecific antibody binding sites. Thereafter, the specimens were incubated with Anti-PLCZ1 Rabbit Polyclonal Antibody (1:100 dilution) for 2 h at 37 °C followed by the incubation with Alexa Fluor 488 Goat Anti-Rabbit IgG (1:200 dilution) for 1 h at 37 °C. Subsequently, the glass slide was washed three times with TL-HEPES-PVA and mounted with VECTASHIELD medium (H-1000, Vectorlabs, Burlingame, CA, USA). Images were obtained by using a fluorescence microscope (BZ-X710, KEYENCE Inc., Osaka, Japan) at ×400 and ×1,000. Totally 300 spermatozoa were observed per sample and percentages of sperm with 3 categories were calculated, including (1) spermatozoa without PLCZ1 fluorescence exhibition, (2) spermatozoa with PLCZ1 distributing at acrosome region only, and (3) spermatozoa with PLCZ1 detected at both acrosomal and equatorial segment regions.

### Experimental design

#### Experiment 1: Monitoring the change of temperature inside frozen straw thawed in water at different temperatures

The change of temperature inside straws of bull semen was observed every two-second interval (a minimum recording interval of the thermometer) during thawing by using a two-channel record thermometer (TNA-140; TASCO Inc., Tokyo, Japan). The first sensor wire of the thermometer was inserted through the cut end of that straw and corked to prevent the semen inside from leaking, and then the straw was immersed back into liquid nitrogen to be frozen again. The second sensor wire was used to monitor temperature of thawing water (39 or 70 °C) in a temperature control bath. Recording of temperature change was started just before the straw in liquid nitrogen was plunged into water at 39 or 70 °C and continued until the temperature inside the straw reached at least 39 °C. The recording was replicated eight times with different straws of frozen semen in each experimental group.

#### Experiment 2: Evaluation of post-thawed characteristics of frozen bull spermatozoa thawed in water at different temperatures

The straws containing bull spermatozoa were rapidly thawed in water at 70 °C for 8 seconds and promptly followed by stabilization in water at 39 °C for 52 seconds (RT70; according to the results in experiment 1). As controls, other straws were conventionally thawed and preserved in water at 39 °C for 60 seconds (CT39). Thawed sperm suspension was diluted in TL-HEPES-PVA pre-warmed at 39 °C and washed once (700×*g,* 3 min, 39 °C) (Athurupana et al., 2015). The precipitate re-suspended in pre-warmed TL-HEPES-PVA (1x10^8^ cells/mL, called a sperm sample) was evaluated after incubating at 39 °C for 5 min (termed as 0 h after thawing).

Basically, a 2x4 factorial design (two thawing methods with CT39 and RT70; frozen semen from 4 different bulls) was applied to examine the effects of thawing methods, bulls, or interaction between thawing methods and bulls on viability, MMP, motility just after thawing (0 h) and after culture for 3 h at 39 °C in TL-HEPES-PVA supplemented with 6 mg/mL BSA. Intracellular ROS level of spermatozoa, acrosome integrity, and distribution of PLCZ1 were compared between thawing methods just after thawing. Experiments were replicated 6 times (different frozen straws from each bull semen) in all experimental groups.

### Statistical analysis

Adapted to the experimental design, statistical analyses were carried out by one-way or two-way ANOVA followed by Turkey’s multiple range tests using GraphPad Prism 8.3 statistical software (GraphPad Software Inc., San Diego, CA, USA). Before the statistical analysis, to fit a normal distribution, all the percentage data for the experiment were subjected to arc-sine transformation if there were values >90% or <10%, then they were posteriorly transformed back into percentages for the tables and figures. The continuous-valued data such as VSL, VCL, VAP, ALH and BCF were transformed by logarithmic transformations if there were non-normal distributions. All results were expressed as mean ± SEM (standard errors of the mean). Differences were considered significant at *P* < 0.05.

## Results

### Monitoring the change of temperature inside frozen straw during thawing

As shown in Table 1, the warming rates inside the straw within 10 seconds were significantly faster (*P* < 0.01) when frozen semen was thawed at 70 °C than 39 °C, without those at 4 and 10 seconds after the start of thawing. The estimated average time for temperature inside the straw to reach from -196 to 0, 15, 37 and 39 °C was significantly faster (*P* < 0.01) when frozen semen was thawed at 70 °C than 39 °C (Table 1). Since the temperature inside the straw thawed in 70 °C water reached 39 °C in 8.2 seconds, the method of stabilizing with 39 °C water after thawing at 70 °C for 8 seconds was adopted in the following experiments.

**Table 1:** Average temperature and warming rates (mean ± SEM) inside the straw during thawing.

**Time (sec) after plunged into thawing water**	**Temperature of water in which frozen straws were thawed at**			
	**39 °C**		**70 °C**	
	**Temperature (°C) in straw**	**Warming rate (°C/sec)**	**Temperature (°C) in straw**	**Warming rate (°C/sec)**
0	-196.0	-	-196.0	-
2	-45.2 ± 5.6^B^	75.4 ± 2.8^b^	-28.9 ± 3.9^A^	83.6 ± 1.9^a^
4	-10.8 ± 1.0^B^	17.2 ± 2.3	-5.3 ± 0.9^A^	11.8 ± 1.6
6	-6.3 ± 0.7^B^	2.2 ± 0.6^b^	11.9 ± 2.3^A^	8.6 ± 1.3^a^
8	-4.7 ± 0.4^B^	0.8 ± 0.4^b^	37.2 ± 3.2^A^	12.6 ± 1.7^a^
10	2.2 ± 1.5^B^	3.4 ± 0.7	47.7 ± 2.3^A^	5.3 ± 0.9
12	10.0 ± 2.0	3.9 ± 1.0	-	-
14	20.2 ± 1.8	5.1 ± 1.0	-	-
16	26.3 ± 1.0	3.1 ± 1.2	-	-

**The change sphere of temperature**	**Estimated average time (sec) to take the change of temperature inside straws thawed at**	
	**39 °C**	**70 °C**
-196 to 0 °C	9.6 ± 0.3^A^	5.1 ± 0.2^B^
-196 to 15 °C	13.0 ± 0.4^A^	6.5 ± 0.2^B^
-196 to 37 °C	39.8 ± 0.3^A^	7.6 ± 0.2^B^
-196 to 39 °C	43.8 ± 0.8^A^	8.2 ± 0.3^B^

*Note*: Different small and capital superscripts indicate a significant difference in temperature and warming rate within the same row, respectively (*P* < 0.05, n = 8).

### Evaluation of post-thaw characteristics of frozen bull spermatozoa thawed in water at different temperatures

There were significant differences in percentages of viable and high MMP spermatozoa not only among bulls (*P* < 0.01) but also between CT39 and RT70 (*P* < 0.05) at 0 and 3 h after thawing (Table 2).

**Table 2:** Percentages (mean ± SEM) of viable and high MMP spermatozoa at 0 and 3 h after thawing under different conditions.

**Thawing protocols**	**Bull**	**Viability** **(0 h, %)**	**Viability** **(3 h, %)**	**High MMP** **(0 h, %)**	**High MMP** **(3 h, %)**
CT39	A	57.7 ± 4.2^c^	40.7 ± 0.4^c^	46.9 ± 1.5^d^	41.5 ± 0.4^bc^
	B	73.4 ± 0.4^ab^	69.7 ± 0.7^a^	82.5 ± 1.1^a^	65.1 ± 1.7^a^
	C	65.2 ± 1.9^bc^	54.3 ± 0.6^b^	55.1 ± 2.2^bc^	38.1 ± 0.5^c^
	D	36.5 ± 0.9^d^	27.9 ± 1.3^d^	49.8 ± 2.2^cd^	25.2 ± 1.1^d^
	Total	58.2 ± 3.1	48.1 ± 3.3	58.6 ± 3.1	42.4 ± 3.1
RT70	A	65.1 ± 4.6^bc^	43.2 ± 1.2^c^	52.6 ± 2.2^cd^	45.2 ± 0.5^b^
	B	77.2 ± 0.5^a^	73.9 ± 0.5^a^	83.3 ± 1.2^a^	69.4 ± 1.4^a^
	C	68.8 ± 1.2^ab^	58.8 ± 1.1^b^	61.3 ± 1.6^b^	41.5 ± 1.7^bc^
	D	37.4 ± 0.9^d^	27.5 ± 1.5^d^	47.9 ± 1.9^cd^	26.1 ± 0.6^d^
	Total	62.1 ± 3.3	50.8 ± 3.7	61.3 ± 3.0	45.5 ± 3.3
*P* among bulls		<0.01	<0.01	<0.01	<0.01
*P* between thawing protocols		*<*0.05	<0.01	*<*0.05	<0.01
*P* TPS x bulls		0.62	0.06	0.09	0.44

*Note*: Different small superscripts indicate a significant difference among bulls, respectively (*P* < 0.05, n = 6). *P* values from two-way ANOVA analysis (n = 6) are shown the bottom of the table. CT39: Spermatozoa were conventionally thawed and stabilized at 39 °C for 60 seconds. RT70: Spermatozoa were rapidly thawed at 70 °C for 8 seconds and then stabilized at 39 °C for 52 seconds.

Although all motility parameters examined in this study were significantly different (*P* < 0.05) among bulls (only an exception of VAP at 0 h), some parameters such as the total motility, progressive motility, and VAP were significantly different (*P* < 0.05) between controls (CT39) and RT70 at 0 (Table 3) and 3 h after thawing (Table 4). However, the rapid progressive motility, VSL, VCL, ALH, and BCF of spermatozoa were not different between RT70 and CT39 (Tables 3 and 4).

**Table 3:** Motility parameters (mean ± SEM) of frozen spermatozoa just (0 h) after thawing under different conditions.

**Thawing protocols**	**Bull**	**Total** **motility (%)**	**Progressive motility (%)**	**Rapid progressive motility (%)**	**VSL** **(μm/s)**	**VCL** **(μm/s)**	**VAP** **(μm/s)**	**ALH** **(μm)**	**BCF** **(Hz)**
CT39	A	43.9 ± 2.0^bc^	38.8 ± 2.3^cd^	32.2 ± 2.7^cd^	58.2 ± 4.9^ab^	195.9 ± 6.5^ab^	73.8 ± 1.6	4.0 ± 0.2^abc^	8.8 ± 1.0^b^
	B	69.2 ± 2.2^a^	57.1 ± 3.8^ab^	49.0 ± 3.9^ab^	48.8 ± 5.3^b^	207.0 ± 10.7^ab^	73.2 ± 5.8	4.3 ± 0.2^ab^	6.6 ± 0.4^b^
	C	44.2 ± 1.6^bc^	36.4 ± 1.2^cd^	30.0 ± 1.7^cd^	65.2 ± 5.0^ab^	192.2 ± 9.4^ab^	78.8 ± 4.8	3.3 ± 0.2^c^	11.5 ± 0.2^a^
	D	34.5 ± 1.7^d^	27.8 ± 1.9^d^	22.8 ± 1.7^d^	62.6 ± 2.8^ab^	198.1 ± 3.4^ab^	77.8 ± 2.5	3.9 ± 0.1^abc^	8.4 ± 0.3^b^
	Total	47.9 ± 2.8	40.0 ± 2.5	33.5 ± 2.4	58.7 ± 2.5	198.3 ± 3.9	75.9 ± 2.0	3.9 ± 0.1	8.8 ± 0.4
RT70	A	50.9 ± 2.8^b^	45.1 ± 2.9^bc^	38.1 ± 2.4^bc^	64.8 ± 3.4^ab^	203.1 ± 7.5^ab^	81.0 ± 2.7	4.0 ± 0.2^abc^	9.0 ± 0.9^ab^
	B	75.8 ± 1.7^a^	67.1 ± 4.9^a^	55.0 ± 4.5^a^	52.0 ± 2.3^b^	218.8 ± 4.6^a^	82.9 ± 2.7	4.4 ± 0.2^a^	7.1 ± 0.3^b^
	C	48.6 ± 2.5^b^	42.2 ± 3.0^c^	34.5 ± 2.4^cd^	69.9 ± 3.3^a^	191.6 ± 8.5^ab^	83.6 ± 3.3	3.5 ± 0.1^bc^	11.6 ± 0.7^a^
	D	35.7 ± 1.6^cd^	27.5 ± 1.8^d^	21.9 ± 2.2^d^	58.4 ± 3.2^ab^	184.1 ± 6.7^b^	78.2 ± 4.6	3.5 ± 0.2^bc^	8.4 ± 0.3^b^
	Total	52.8 ± 3.2	45.5 ± 3.3	37.4 ± 2.8	61.3 ± 2.0	199.4 ± 4.2	81.4 ± 1.7	3.8 ± 0.1	9.0 ± 0.4
*P* among bulls		<0.01	<0.01	<0.01	<0.01	<0.05	0.74	<0.01	<0.01
*P* between thawing protocols		<0.01	0.01	0.06	0.36	0.84	<0.05	0.73	0.65
*P* TPS x bulls		0.48	0.38	0.59	0.55	0.34	0.65	0.50	0.97

*Note*: Different small superscripts indicate a significant difference among bulls, respectively (*P* < 0.05, n = 6). *P* values from two-way ANOVA analysis (n = 6) are shown the bottom of the table. CT39: Spermatozoa were conventionally thawed and stabilized at 39 °C for totally 60 seconds. RT70: Spermatozoa were rapidly thawed at 70 °C for 8 seconds and stabilized at 39 °C for 52 seconds.

**Table 4:** Motility parameters (mean ± SEM) of frozen spermatozoa 3 h after thawing under different conditions.

**Thawing protocols**	**Bull**	**Total motility (%)**	**Progressive motility (%)**	**Rapid progressive motility (%)**	**VSL** **(μm/s)**	**VCL** **(μm/s)**	**VAP** **(μm/s)**	**ALH (μm)**	**BCF (Hz)**
CT39	A	29.9 ± 0.8^c^	24.2 ± 1.3^b^	20.9 ± 1.5^bc^	50.4 ± 3.0^ab^	161.1 ± 8.6^ab^	73.2 ± 4.1^ab^	2.9 ± 0.1^ab^	12.5 ± 0.7^ab^
	B	51.4 ± 1.5^b^	42.7 ± 3.0^a^	35.6 ± 3.1^a^	48.6 ± 3.1^ab^	159.8 ± 15.2^ab^	67.3 ± 4.1^ab^	3.3 ± 0.3^a^	12.7 ± 0.7^ab^
	C	28.4 ± 0.9^c^	22.5 ± 0.9^bc^	18.9 ± 1.6^bcd^	56.4 ± 4.8^a^	164.4 ± 11.6^ab^	76.2 ± 4.7^a^	2.5 ± 0.3^b^	15.1 ± 0.6^a^
	D	20.6 ± 1.4^d^	14.2 ± 1.2^d^	10.6 ± 1.6^d^	40.9 ± 3.7^b^	134.5 ± 8.3^b^	61.1 ± 7.3^b^	2.7 ± 0.3^b^	10.8 ± 0.4^b^
	Total	32.6 ± 2.5	25.9 ± 2.3	21.5 ± 2.1	49.1 ± 2.8	154.9 ± 5.8	69.4 ± 2.7	2.9 ± 0.2	12.8 ± 0.4
RT70	A	34.0 ± 1.2^c^	29.2 ± 1.0^b^	24.8 ± 1.8^b^	57.2 ± 7.1^ab^	171.2 ± 11.8^ab^	77.9 ± 5.6^ab^	3.2 ± 0.2^ab^	13.6 ± 1.0^ab^
	B	57.3 ± 2.0^a^	48.9 ± 2.6^a^	38.3 ± 3.2^a^	51.0 ± 7.3^ab^	180.1 ± 6.9^a^	81.6 ± 2.6^ab^	3.5 ± 0.3^a^	13.0 ± 0.3^ab^
	C	30.7 ± 1.1^c^	26.3 ± 1.3^b^	21.6 ± 1.1^bc^	62.6 ± 3.2^a^	161.3 ± 7.0^ab^	80.0 ± 3.7^a^	2.6 ± 0.2^b^	14.2 ± 0.5^a^
	D	21.8 ± 1.2^d^	15.4 ± 1.9^cd^	12.3 ± 2.1^cd^	46.6 ± 6.5^b^	151.7 ± 5.4^ab^	67.0 ± 6.0^b^	2.6 ± 0.2^b^	11.0 ± 0.6^b^
	Total	36.0 ± 2.8	29.9 ± 2.7	24.3 ± 2.2	54.3 ± 3.2	166.1 ± 4.4	76.6 ± 2.5	3.0 ± 0.1	12.9 ± 0.4
*P* among bulls		<0.01	<0.01	<0.01	<0.05	<0.05	<0.05	<0.01	<0.01
*P* between thawing protocols		<0.01	<0.01	0.07	0.15	0.12	<0.05	0.71	0.72
*P* TPS x bulls		0.30	0.55	0.96	0.97	0.64	0.70	0.96	0.54

*Note*: Different small superscripts indicate a significant difference among bulls, respectively (*P* < 0.05, n = 6). *P* values from two-way ANOVA analysis (n = 6) are shown the bottom of the table. CT39: Spermatozoa were conventionally thawed and stabilized at 39 °C for 60 seconds. RT70: Spermatozoa were rapidly thawed at 70 °C for 8 seconds and then stabilized at 39 °C for 52 seconds.

**Figure 1:**
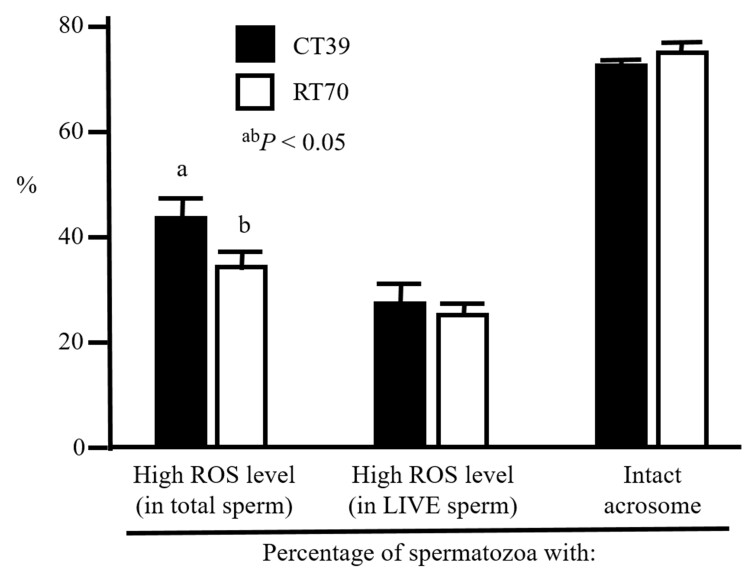
Percentages of spermatozoa with high ROS levels and intact acrosome just after thawing at different conditions. Different superscripts indicate a significant difference in each parameter (*P* < 0.05, n = 6). CT39: Spermatozoa were conventionally thawed and stabilized at 39 °C for totally 60 seconds. RT70: Spermatozoa were rapidly thawed at 70 °C for 8 seconds and then stabilized at 39 °C for 52 seconds.

As shown in Figure 1, the percentage of spermatozoa with high ROS level was significantly lower (*P* < 0.05) in RT70 than CT39, whereas there were no differences in the percentage of “live” spermatozoa with high ROS level. Furthermore, there were no significant differences in the percentage of spermatozoa with intact acrosome between two thawing conditions examined (Figure 1).

**Figure 2:**
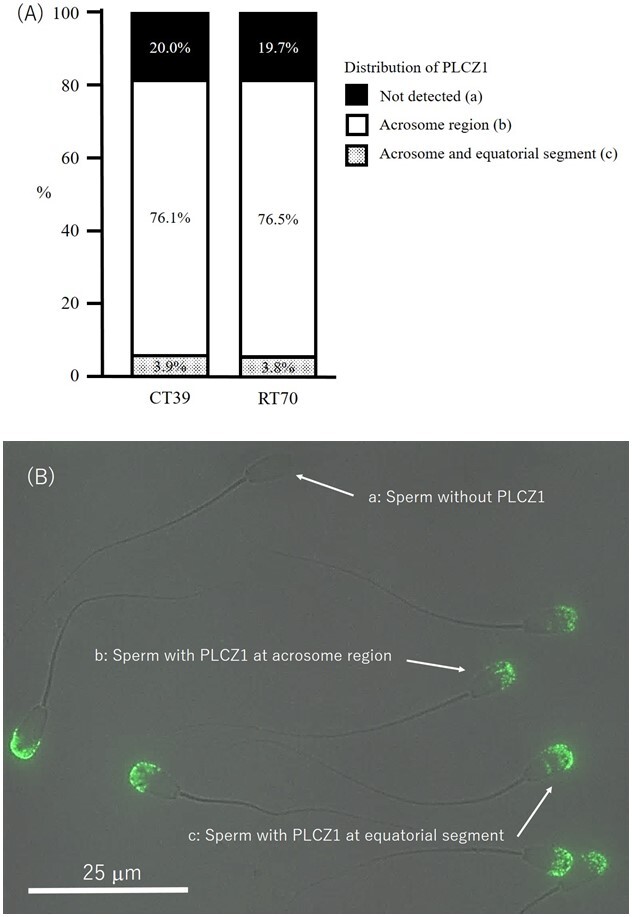
(A) The distribution of PLCZ1 in spermatozoa thawed at different conditions (n = 6). The numbers in the bars indicate the percentages of spermatozoa in each criterion of PLCZ1 distribution. CT39: Spermatozoa were conventionally thawed and stabilized at 39 °C for 60 seconds. RT70: Spermatozoa were rapidly thawed at 70 °C for 8 seconds and then stabilized at 39 °C for 52 seconds. (B) Image including 3 fluorescence patterns (a, b and c) of PLCZ1 distribution in spermatozoa was taken at ×1,000. Bar indicates 25 μm.

Regardless of the thawing condition, fluorescence of PLCZ1 was detected at the acrosome region (76.1-76.5%; Figure 2) of the majority of spermatozoa. In a few parts of spermatozoa (3.8-3.9%), the fluorescence was detected at both the acrosome and equatorial segment regions, whereas the others (19.7-20.0%) were not detected the presence (Figure 2). However, these percentages were not different between RT70 and CT39.

## Discussion

For long decades, frozen semen of domestic animals has been mostly thawed around body temperature, 37-39 °C (Correa et al., 1996; Mehmood et al., 2017; Ogata et al., 2022; Ömür, 2022). Since the harmful temperature zone in frozen spermatozoa is often occurred critically, due to the deleterious recrystallization of intracellular ice, between -60 and 0 °C, around the melting point switching from glassy to liquid phase, during thawing, it needs to pass through the dangerous range as soon as possible. In the present study, when frozen semen was thawed in water at 70 °C, the temperature inside the straw rose from -196 to 0, 15, 37, or 39 °C significantly faster, as compared with thawing at 39 °C. Therefore, when frozen semen was thawed at 39 °C, as compared with thawing at 70 °C, spermatozoa inside the straw seem to expose longer to the dangerous temperature range and also to the low temperatures outside the physiological range. Furthermore, it has been well known that changes in temperature, osmotic stress, toxicity of glycerol and formation of ice crystals during not only freezing but also thawing processes make damage spermatozoa (Hammerstedt et al., 1990; Samper et al., 1991; Lessard et al., 2000; Cerolini et al., 2001), resulting in less sperm viability (Royere et al., 1996). In the present study, since both frozen semen thawed at 39 and 70 °C was not diluted with the fresh medium until totally 60 seconds after thawing, the condition exposed to osmotic stress and glycerol was similar in both groups. Therefore, two thawing rates in the present study may make differences in ice crystal reformation within the semen and the effects of exposure to non-physiological temperatures on thawing spermatozoa.

Rapid thawing did not seem to have a firm evaluation, with a report that viability and acrosome integrity of bull spermatozoa did not differ between thawing at 60 and 37 °C (Al-Badry, 2012) while another report demonstrated that rapid thawing at 65-70 °C maintained higher viability and motility of frozen bull spermatozoa compared with thawing at 35 °C (Lyashenko, 2015). In the present study, we demonstrated that not only the viability but also percentage of spermatozoa with high MMP were significantly different between CT39 and RT70 at 0 and 3 h after thawing. Since we also observed that the percentage of spermatozoa with intact acrosome did not differ between CT39 and RT70, there does not appear to be a difference in effect between rapid and conventional thawing methods examined that would damage the acrosome membrane. Furthermore, although the percentages in “live” cells with high ROS level were not different after thawing at different temperatures, those of all spermatozoa were significantly lower in RT70. These results suggest that rapid thawing method maintains the viability of frozen bull spermatozoa rather than conventional thawing at 39 °C, and conventional thawing may induce more oxidative stress related to ROS production in the spermatozoa.

Our results on CASA evaluation of frozen semen derived from four bulls revealed that the percentages of motility (total and progressive motility) and VAP at 0 and 3 h after thawing were significantly different between CT39 and RT70, consistent with the previous results (Lyashenko, 2015), but was contrasted with the other ones (Al-Badry, 2012; Yilmaz et al., 2019). As described above, furthermore, since the percentage of spermatozoa with high MMP was also significantly different between CT39 and RT70 at 0 and 3 h after thawing, our results on sperm motility could be speculated to reflect positively the state of mitochondria in spermatozoa thawed under RT70 condition.

Furthermore, although we could detect the presence of PLCZ1 at the acrosome region, where consistent with a previous study (Kasimanickam et al., 2012), in the majority of frozen-thawed spermatozoa, we observed that PLCZ1 distribution in spermatozoa was not different between spermatozoa thawed in CT39 and RT70. Previous studies reported that the freezing-thawing process could change expression of important proteins of spermatozoa (Rezaie et al., 2021). PLCZ1 is a kind of soluble cytosolic protein (Nomikos et al., 2013), and is affected by cryopreservation (Kashir et al., 2011), because during cryopreservation, membrane lipids are damaged, leading to instability of the membrane and reduction of many proteins, which are important for sperm fertility (Hezavehei et al., 2021). Combining the evidence that the percentage of spermatozoa with intact acrosome did not differ between CT39 and RT70, therefore, our results suggest that the distribution of PLCZ1 seems to be similarly maintained in spermatozoa under both thawing conditions we examined.

## Conclusion

In conclusion, rapid thawing (RT70) rather than conventional thawing condition (CT39) is recommended to make significant differences in the viability, motility, and MMP of frozen bull spermatozoa. The beneficial effects of rapid thawing could be due to shorter exposure to temperatures from -196 °C to the physiological temperature range, and consequently less damage of the biological membrane associated with mitochondrial health.

## Data Availability

The data support the findings of the current study are available from the corresponding author upon reasonable request.
